# Image-based modelling of lateral magma flow: the Basement Sill, Antarctica

**DOI:** 10.1098/rsos.161083

**Published:** 2017-05-31

**Authors:** Nick Petford, Seyed Mirhadizadeh

**Affiliations:** Faculty of Arts, Science and Technology, University of Northampton, Northampton, UK

**Keywords:** magma, Antarctica, igneous intrusion, fluid dynamics, rheology

## Abstract

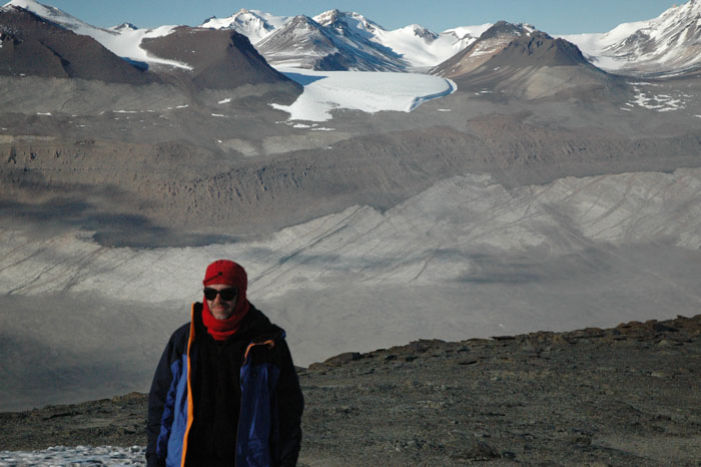

The McMurdo Dry Valleys magmatic system, Antarctica, provides a world-class example of pervasive lateral magma flow on a continental scale. The lowermost intrusion (Basement Sill) offers detailed sections through the now frozen particle microstructure of a congested magma slurry. We simulated the flow regime in two and three dimensions using numerical models built on a finite-element mesh derived from field data. The model captures the flow behaviour of the Basement Sill magma over a viscosity range of 1–10^4^ Pa s where the higher end (greater than or equal to 10^2^ Pa s) corresponds to a magmatic slurry with crystal fractions varying between 30 and 70%. A novel feature of the model is the discovery of transient, low viscosity (less than or equal to 50 Pa s) high Reynolds number eddies formed along undulating contacts at the floor and roof of the intrusion. Numerical tracing of particle orbits implies crystals trapped in eddies segregate according to their mass density. Recovered shear strain rates (10^−3^–10^−5^ s^−1^) at viscosities equating to high particle concentrations (around more than 40%) in the Sill interior point to shear-thinning as an explanation for some types of magmatic layering there. Model transport rates for the Sill magmas imply a maximum emplacement time of *ca* 10^5^ years, consistent with geochemical evidence for long-range lateral flow. It is a theoretically possibility that fast-flowing magma on a continental scale will be susceptible to planetary-scale rotational forces.

## 1. Introduction

While significant progress has been made in quantifying the flow properties of silicate melt in terms of viscosity and density over a wide compositional range [1–5], a fundamental treatment that captures simultaneously the rheology and flow behaviour of magma as a fluid-particle suspension remains elusive. This lack of understanding of what may appear at first glance a relatively trivial problem arises from the fact that magmas are complex multiphase flows with time- and rate-dependent properties. Especially problematic is that those factors governing the flow of magma, including heat transfer, phase transitions, coupled deformation of both solid and fluid phases, seepage phenomena and chemical processes, occur simultaneously and interdependently. Of critical importance is that for multiphase materials such as magma, the flow properties, and by inference the types of structures likely to be formed in cooling magma, are macroscopic properties derived from the suspension microstructure [6–11]. It is this microstructure–viscosity connection that defines the complex rheological properties of magma, which in turn controls its overall rate and style of emplacement on and within the Earth [12–14]. More generally, a better understanding of the motion of particle–fluid mixtures is important for improved understanding of how (and on what timescales) enigmatic structures that occur on a local scale including layering, grading, tubes and pipes form in magmatic systems [15–17].

Many of these features are observed in the Basement Sill, Antarctica, a well-documented mafic intrusion with excellent field exposure [18]. Using data obtained from the field including digital images of the contact geometry, metrics on crystal size and mineralogy, and determinations of melt viscosity based on published data, we have built a numerical model that simulates the local flow behaviour of the Sill magmas.

Our motivation for the study is threefold. First is simply to show that it is possible to use field data (digital photographs) of naturally occurring geological features to make image-based numerical models in ways used successfully in other disciplines [19,20]. Second, to use the flow models to evaluate past emplacement conditions (hindcasting) and gain new insight into the origins of crystal accumulations and layering observed in the Sill. Last is to use the fluid dynamical model to corroborate geochemical evidence for long-range (continental scale) lateral flow and emplacement of the Ferrar magmas in Antarctica on geologically short (less than 1 Ma) timescales. While acknowledging that any model of this kind involves inevitable simplifications, our intention is to show that at least some aspects of the complex flow patterns that form on a scale of hundreds of metres can be quantified within limitations general to all numerical models (non-uniqueness and uncertainties around input variables).

### 1.1. Basement Sill and surrounding geology

**Figure 1.**
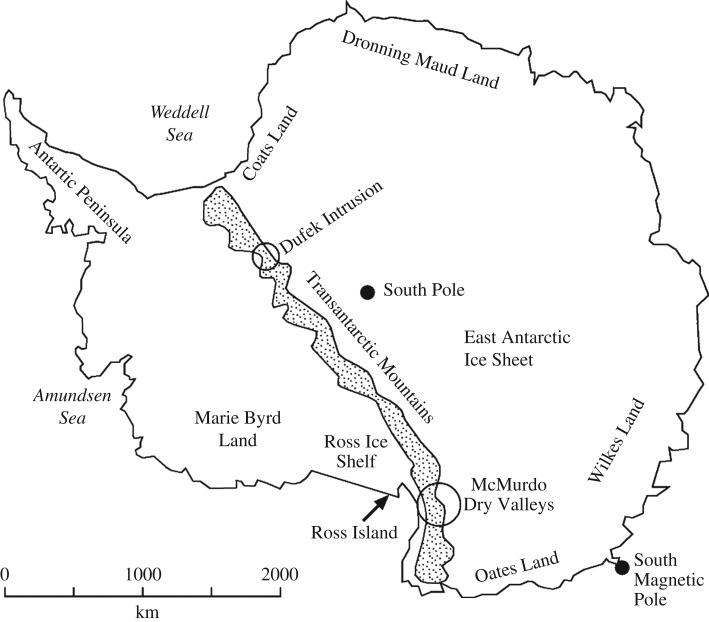
Map of Antarctica showing key locations including the Transantarctic Mountains, Dufek Intrusion, Ross Island (McMurdo Base) and south magnetic pole. The Dry Valleys region is circled.

**Figure 2.**
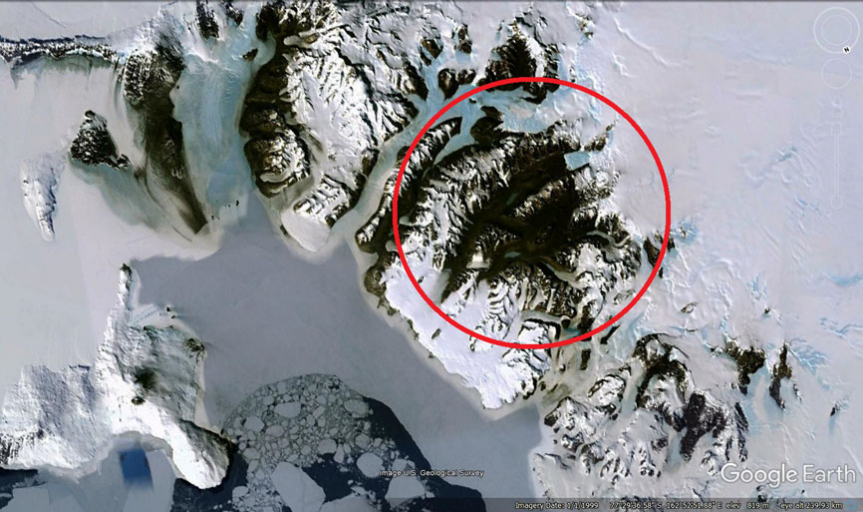
Satellite view of Victoria Land showing the Dry Valleys region (circled), part of the Eastern Transantarctic Mountains, the Ross Ice Shelf and McMurdo Sound. Attribution: UG Geological Survey, PGC/NASA, Data LDEO-Columbia, NSF, NOAA Data SIO, US Navy, NGA, GEBCO.

**Figure 3.**
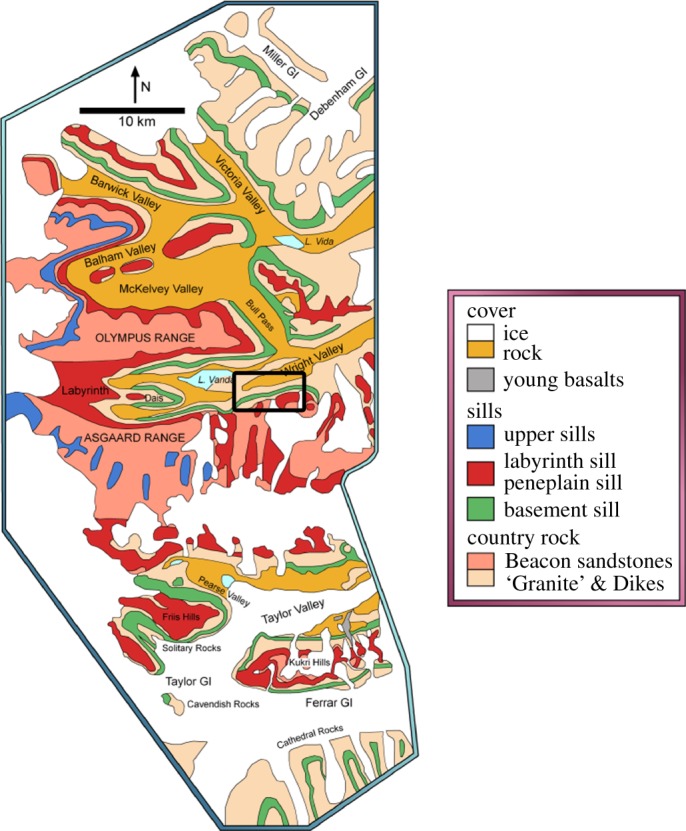
Map of field area. The section of the Basement Sill (green) analysed in this study crops out in the southern wall of the Wright Valley, bounded by the parallels 77°30′ and 77°40′ and meridians 161°–162°. Black rectangle shows study location. (Adapted from [18], AGU, with permission.)

**Figure 4.**
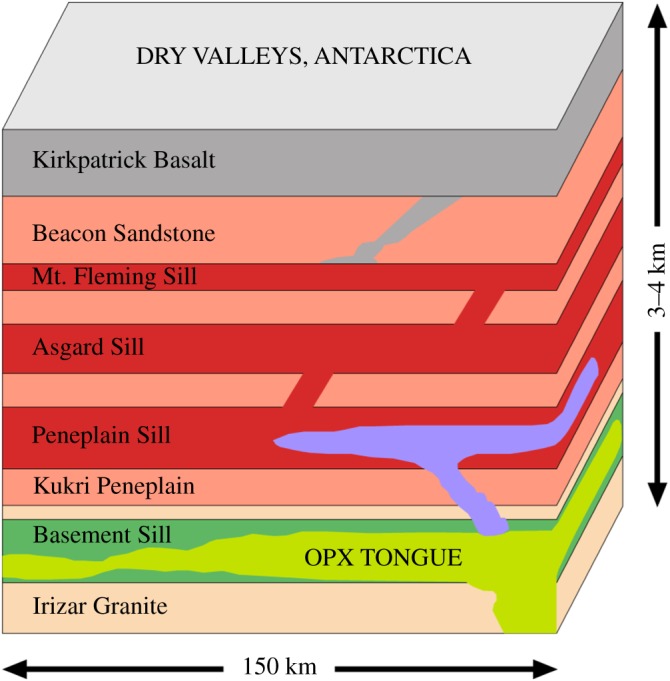
Section showing a stylized vertical stack comprising the intrusive units and country rocks of the Dry Valleys. The surface Kirkpatrick flood basalts are contemporaneous with hyperbassal magmatism. The intrusion sequence was most likely from the top down, with the Basement Sill including the layered Opx tongue the youngest and most mafic, confined entirely to basement rock. (Adapted from [18], AGU, with permission.)

Located towards the southern termination of the Transantarctic Mountains, the massive dolerite sills stretching between Victoria Land and the Beardmore Glacier (figure 1), drew early attention from geologists on both the Scott Discovery 1901 and Shackleton Nimrod 1907–1909 expeditions to the South Polar Region [21,22]. The dolerite rocks formed during the break-up of Gondwana and the Dry Valleys region (figure 2) in the vicinity of McMurdo Bay, Victoria Land, preserves a virtually unfaulted western flank of the former supercontinent [22–24]. The system covers an area of some 5000 km^2^ with the sills dipping 2–5° to the west. Forming part of the Jurassic Ferrar dolerite Large Igneous Province (LIP; figure 3), the complex preserves a world-class example of a multiple, magmatic plumbing system. In detail, The McMurdo Dry Valley dolerites (figure 4) comprise a vertical stack of four interconnected sills linked to surface flows of the Kirkpatrick flood basalts [25–27], dated at about 180 Ma [28].

### 1.2. Basement Sill geology

The lowermost intrusion, the Basement Sill, with an estimated minimum volume of 1050 km^3^ offers unprecedented exposure through a frozen magmatic slurry (a congested melt-particle mixture), comprising abundant Opx pheoncrysts [18,29]. The overall geometry of the axially confined slurry is tongue-like, with the Sill margins relatively aphiric [26]. The tongue has a wide range of structures, including grading, layering and silicic melt segregation in its upper parts. It is noteworthy that features long recognized in classical layered intrusions and attributed to crystal settling formed here on a timescale governed by the local cooling rate of 300 m thick intrusion [27]. Along with Opx, clinopyroxene and plagioclase form the major phases with minor quartz and alkali feldspar. Silica contents range between 51 and 56 wt% [27]. Bedard *et al.* [29] provide a highly detailed description of the Sill geology, including field relations, petrography and mineral chemistry. It is likely that the intrusion is multistage, with initial emplacement of a relatively aphyric magma reaching dimensions of several hundred metres width before a second phase which included injection of the Opx magmatic slurry into the pre-existing Sill [27,29].

This model cannot predict the detailed field relations or unique crystal distributions seen in the field. Instead we assume a single pulse of magma emplaced instantaneously. However, it is possible to gain some insight into the intrusion process by focusing on selected areas of the Sill geometry and using field data including measured crystal size range and mineralogy as a guide. Thus, the lower marginal zone (LMZ) is of interest as this part of the intrusion is the most primitive in composition and used to constrain the initial melt viscosity (see §2.1). The LMZ itself is a thin (0.1 m) aphanitic chilled margin, grading upwards into the lower and middle zones (LZ and MZ, respectively), that includes websterites, pyroxenites and rhythmically layered leuco-gabbros comprising the Opx tongue. The mid to upper sections (upper zone and upper marginal zone) contain gabbronoritic and anorthositic pipes, veins and felsic segregations. These structures cut across igneous layering and were formed towards the end of the emplacement phase [27].

**Figure 5.**
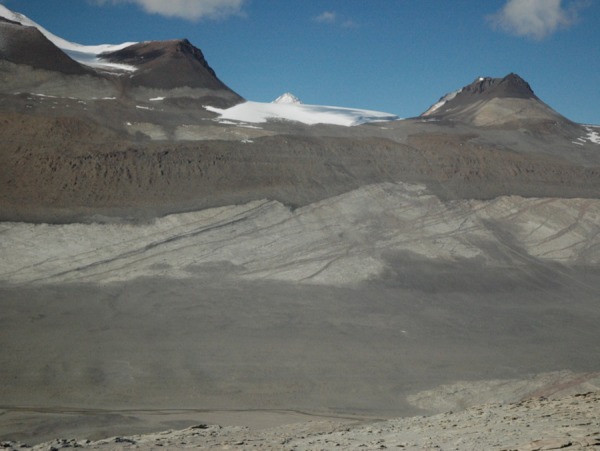
Basement Sill in the South Wall of the Wright Valley at Bull Pass (cf. figure 4) intruding Late Ordovician age Granite Harbour basement rock [27]. Chilled margins at the base (LZ) and layering in the central part of the Sill (Opx tongue) are visible. Note the emplacement style controlled locally by pre-existing basement fractures help create an undulating contact with country rock. From this perspective magma emplacement occurred E–W (left to right in the image) down a shallow 2–5° regional slope. The numerical model of magma flow is constructed around a digitized version of this image using a bespoke finite-element mesh corrected for angular distortion.

Throughout the Sill, the absence of cryptic mineral variations has been used to argue against sequential crystal setting and accumulation [18,29]. Instead the preferred model is that the crystal load was injected as a single (mostly orthopyroxene + plagioclase) mixture. Given this, the fluid dynamical model presented here has some bearing on the types of geological features seen in the field including segregation structures and layering. An equivalence is drawn between these structures and shear-driven dilatant zones in granular slurries in §4.3. In particular, we argue the observed magmatic layering in parts of the Sill interior is the geological expression of shear-thinning in a congested (greater than or equal to 40% solids) magma slurry. The Basement Sill is further noteworthy as the only Ferrar sill in the Dry Valleys to intrude into granitic basement rock, the overlying sills confined exclusively to sandstones of the Beacon Supergroup [26,27]. Importantly for this study, the nature of its contact with granitic basement is controlled by joint systems in the granite [27], resulting in a transgressive, step-like interface (figure 5). This structural irregularity has implications for the flow of magma in the LZ and is discussed in detail later.

## 2. Material and methods

Image-based computer modelling can be a powerful method of obtaining data on flow velocities and other derivative properties of intruding magma using data obtained from the field. We begin by presenting the initial results of a two-dimensional numerical model employing a generalized form of the Navier–Stokes equations (viscous stress tensor formulation) in time-dependent mode (table 1). The numerical model is imaged-based, meaning that the finite-element mesh and bounding geometry is constructed to scale from photographs taken in the field (e.g. figure 5). Field images were digitized and imported into the software via a Matlab routine. The fact that the final observed intrusion geometry is unlikely to be that during active magma emplacement is not lost on us [39]. However, guided as it is by the preserved shape of the intrusion, the model captures a level of realism beyond assuming magma flow between two infinite parallel plates [40–42].

**Table 1.** Summary of variables and parameter values used to model magma flow.

variable or expression	value or range	references
calculated melt viscosity (dynamic) at 1200°C	33 Pa s	[5,27]
PDE formulation	*boundary conditions*	model input
Navier–Stokes (viscous stress tensor)	inflow velocity **u** = **u**_0_	
ρ(∂u/∂t)−∇⋅[η(∇u+(∇u)T)]+ρ(u⋅∇)u+∇p=F	outflow/pressure *p* = p_0_	
∇⋅u=0	no slip **u** = **0**	
	*initial conditions*	
	magma density *ρ*	
	magma viscosity *η*	
magma viscosity (dynamic), where *η* = *η*_0_/(1−R*ϕ*)^2.5^	10^2^ Pa s, *ϕ* = 30%	[30]
*ϕ* = solids by volume	10^3^ Pa s, *ϕ* = 56%	
R = 1/maximum packing factor π/3√2	10^4^ Pa s, *ϕ* = 67%	
*η*_0_ = 33 Pa s		
magma velocity at 33 Pa s	0.15 m s^−1^	model output
magma viscosity (kinematic) = *η*/*ρ*		model output
magma density (*ρ*), kg m^−3^	2500–2950, ref = 2900	[27]
crystal (particle) size mm	1.0–10.0	[29,31,32]
particle density range kg m^−3^	2000–5000	[27,31]
	(Opx 3209–3900)	
	(Plag 2500–2900)	
	Fe-Ti oxides (4500–5000)	
average shear rate ($\dot{\gamma }$) s^−1^	$\dot{\gamma }=10^{-3},$ *ϕ* = 30%	model output and [33–36]
	$\dot{\gamma }=10^{-4},$ *ϕ* = 56%	
	$\dot{\gamma }=10^{-5},$ *ϕ* = 67%	
intrusion temperature	1250 Celsius	[29]
local pressure gradient, Pa m^−1^	0.5	model output
intrusion depth	4 km	[18]
intrusion length L (minimum)	100 km	[18]
intrusion width (m)	50–300, (model = 80)	[18,27]
intrusion area (minimum)	10 000 km^2^	[27]
Coriolis parameter 2*ω*	1.46 × 10^−4^ s^−1^	[37]

The numerical model was built and solved using Comsol Multiphysics v. 5.1 in pipe flow and particle tracing mode with magma density and viscosity supplied as variables (see §2.1). The model dimensions correlate with a section of the Basement Sill in the Bull Pass area 80 m in the vertical and 200 m in the horizontal. This covers just a small part of the overall structure but is large enough to capture flow effects from a field perspective. The models presented are dynamic (involving both forces and kinematics) but do not consider thermal effects or changes in bulk density. In this sense, they are flawed but nonetheless allow insight into fluid particle interactions as first-order effects.

### 2.1. Viscosity and density

Input viscosity and density ranges used in this study are given in table 1. Key considerations in the model are the viscosity of the melt phase and how the effective viscosity changes with increasing crystal load. The initial melt viscosity was estimated from Giordano *et al.* [5] using whole rock chemical analysis reported by Hamilton [27, p. B31, table 1] for the chilled margin (LMZ) of the Basement Sill. Mineral thermometry implies emplacement temperatures in the range 1200°C < *T* < 1280°C [27]. In total, 1200°C recovers a calculated melt viscosity of log 1.6 Pa s (approx. 33 Pa s) for 1 wt% H_2_O. This is higher than the 10 Pa s used to model particle settling times in the Basement Sill magma [29], but similar to the reported suspension viscosity for 20% entrained plagioclase crystals by volume [29]. In passing we note the calculated melt viscosity at high temperature is similar to experimental results for other natural basaltic melts undergoing small amplitude oscillatory shear (28 Pa s, *T* = 1200°C, [43]). An important facet of our model was to explore the stepwise effect of increasing magma viscosity on overall flow behaviour and emplacement timescales. Parametrization of the evolving suspension (effective) viscosity from 1 to 10^4^ Pa s with increased particle loading is shown in table 1 for a fixed melt viscosity of 33 Pa s. We consider this viscosity range the most appropriate for the case in question but note it is possible to assign any reasonable value of magma viscosity to the model runs as an input variable.

Dry powder whole rock densities of 2.80–2.95 g cm^−3^ [27] from the LMZ were used to constrain the density of the melt phase in the model (2500–2900 kg m^−3^). Our chosen model range of particle diameters (1.0–10.0 mm) matches closely the observed distribution in crystal size for the Basement Sill rocks [26,27,29]. Model particle densities of 2000–5000 kg m^−3^ capture the density range in the major mineral phases comprising the Sill (plagioclase 2600–2800 kg m^−3^, orthopyroxene, approx. 3000–4000 kg m^−3^).

The COMSOL files used in this model including full boundary and initial conditions can be accessed at https://www.comsol.com/community/exchange/491/. Alternative model runs capturing sensitivity analysis around input variables are available at the Dryad Digital Repository.

### 2.2. Particle tracing

We were interested in finding out more about how crystals might interact macroscopically in the flow field during emplacement, a feature of significant interest but one hard to unravel using conventional field mapping techniques. To this end, a key aspect of the model is the ability to trace particles (proxies for crystals) during flow simulations and monitor how they interact in the shear field relative to each other as a function of particle density. Particle tracing in the model follows Newton's second law d**P**/d*t* = d(*m***v**)/d*t* = **F**, where the force **F** depends on the background field, in this case the fluid velocity **F = F(v)**. **P** is the linear momentum of a particle (crystal in the magma) with mass *m* (**P** = *m***v**). Flow is assumed left to right along strike of the intrusion (in the field E–W) and is supported by field data for magma flowing along a shallow regional gradient towards the west [18]. For simplicity, we assume both the Opx slurry interior and surrounding melt (now largely chilled margin) coexist so that both are flowing simultaneously in the geometry preserved in the field. Finally, we have extended the flow results locally into the third dimension to investigate the types of interactions between particles perpendicular to the mean direction of flow at the Sill country rock interface.

### 2.3. Flow velocity

It is of considerable importance in the model to establish how fast the magma was flowing for a given viscosity and other fixed variables. In lateral dyke propagation models of magma, flow is generally assumed horizontal along a boundary of level neutral buoyancy (LNB) in the crust, controlled by the local pressure gradient d*w/*d*x* (e.g. [44,45]). However, in many volcanic rift zones, magma propagation follows topographical slopes in the subsurface, meaning in effect the fluid is flowing ‘downhill’ [46]. This appears to be the case for the Basement Sill, which has a regional dip (*α*) of approximately 5° [18]. Taking the average model Sill width (*w*) of 80 m gives a local velocity of approximately 0.15 m s^−1^ for the calculated melt viscosity (*η*) of 33 Pa s. Mean flow velocities of this order are comparable broadly with other estimates from basaltic systems where lateral flow of magma has occurred. For example, Macdonald *et al.* [47] calculate average flow velocities of 0.2–5.0 m s^−1^ for lateral emplacement of the Cleveland Dyke in the British Tertiary Volcanic Province, while Fialko & Rubin [46] report values of 0.3 m s^−1^ for a 1% slope along the Juan de Fuca ridge, see also [48]. For comparison, while silicic (higher viscosity) magmas move more slowly, Kerr and Lister [49] estimated a lateral emplacement time for the 100 km-long Tenant Creek porphyry of about a year. Because lateral flow is driven by local pressure gradients [42,44], the calculated flow velocity was used to derive a mean pressure gradient of approximately 0.5 Pa m^−1^ (see [47,50] for other estimates of local pressure gradients in basaltic sills).

## 3. Results

### 3.1. Two-dimensional flow field

**Figure 6.**
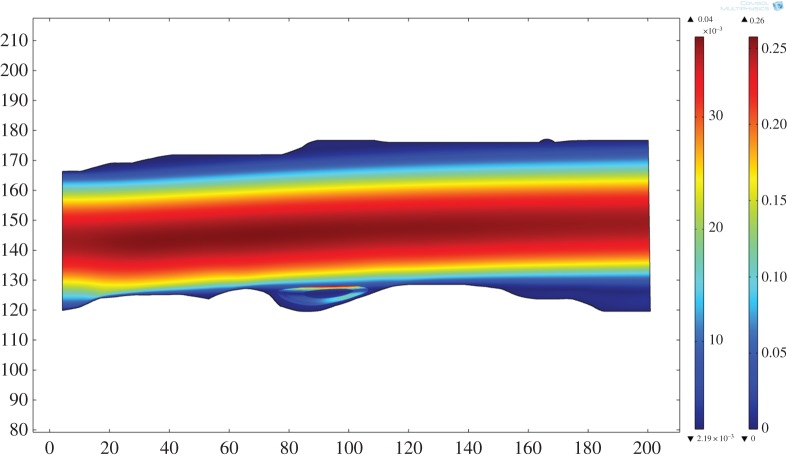
Two-dimensional model simulation of magma flow in the Basement Sill solved in the original (field) geometry. The model recovers the unique surface velocity field for a magma viscosity of 10 Pa s and density of 2500 kg m^−3^. As expected from standard analytical solutions for Poiseuille-type flow, velocities are fastest in the centre (approx. 0.25 m s^−1^) and decrease parabolically towards the margins of the intrusion [51]. Note the eddy formed at the lower boundary. Axis units in m.

As an example, visualization of the two-dimensional flow field for the Basement Sill calculated in the original field geometry is shown in figure 6 for a melt viscosity of 10 Pa s and average density of 2500 kg m^−3^. The velocity profile is (as expected) parabolic with a maximum flow velocity of 0.25 m s^−1^. Despite the rather large number of simplifications made, the model provides some useful insight. For example, assuming flow is continuous (that is, a ready supply of magma is available at depth), the magma could travel the approximately 150 km length of the exposed Sill in approximately 10 days. For comparison, Green *et al*. [48] measured dyke propagation rates based on combined GPS and seismic data in Iceland of approximately 4.6 km d^−1^. The timescales and implications for long-range lateral magma transport using the model are discussed in more detail in §4.4.

**Figure 7.**
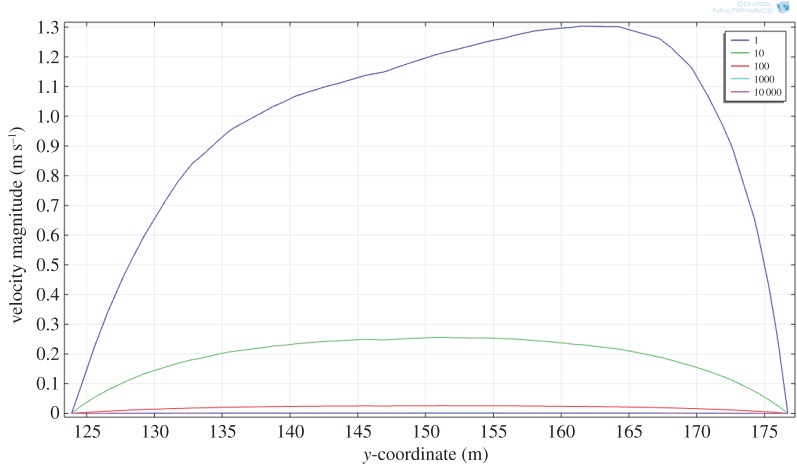
Effects of increasing melt viscosity on the cross-sectional (*y*-coordinate) flow velocity taken at the same mid-point of the Sill (*x* = 120, figure 6). Velocities range from approximately 1.3 m s^−1^ (viscosity 1 Pa s) to 3 × 10^−4^ m s^−1^ (viscosity 10^4^ Pa s).

**Figure 8.**
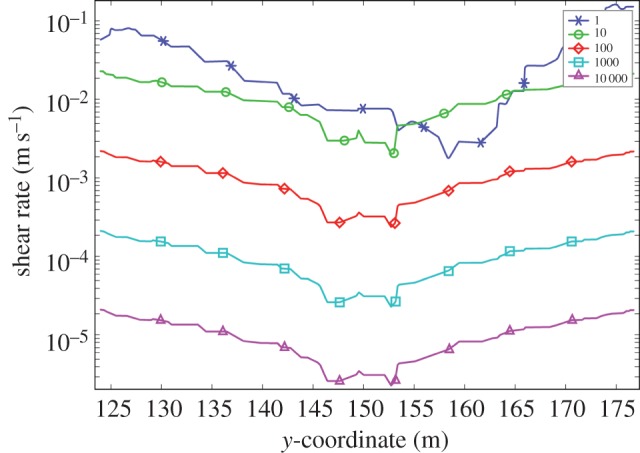
Variation in cross-sectional shear rate ($\dot{\gamma }\;{\text{s}}^{-1}$) for a range of magma viscosities (Pa s) at position *x *= 120.

It is also possible to calculate the effect of increasing magma viscosity on the overall flow rate. We have chosen to do this by including explicitly the effects of increasing particle concentrations on the effective viscosity (table 1). Figure 7 shows the result of increasing the magma viscosity from 1.0 to 10^4^ Pa s by treating it as a suspension with 30% to around 60% solids (e.g. [4,12,13,30]). The upper limit corresponds to the later stages of magma emplacement where crystallization has evolved the magma to a congested state. Not surprisingly the effect is pronounced. As expected, the maximum flow velocity drops as the viscosity of the magma increases, from approximately 1.3 m s^−1^ (melt phase only at 1 Pa s) to approximately 3 × 10^−4^ m s^−1^ for a congested slurry (10^4^ Pa s, 67% solids). Recovered shear rates also vary with magma viscosity. Shearing in congested materials during flow can result in layering due to shear thinning [52], leading to the idea this could explain some of the structures seen in the Basement Sill Tongue [12,29]. The cross-stream shear rate ($\dot{\gamma }$) over the model viscosity range is shown in figure 8, where $10^{-1}<\dot{\gamma }<10^{-5}\;{\text{s}}^{-1}$. The idea that shear rate can induce layering in congested magma through shear thinning is discussed with reference to the LZ websterites in §4.3.

### 3.2. Eddy flow

Of interest are regions in the flow model defined by local and discrete patterns of circulation at low magma viscosity. These simulated patterns form in the base (and roof) of the model intrusion at undulations along the contact between the Sill and country rock corresponding to the lower and upper zones of the intrusion. This pattern of flow can be seen in figure 6. On closer inspection of the velocity field, it is clear that these regions are defined by rotation and counterflow in the melt streamlines. The streamlines are thus defining standing eddies that form in the model where the flow encounters uneven boundaries. The eddies are attached (locally confined) and geometrically similar to vortices common to fluids moving past an obstacle or overstep where flow separation leading to instability has occurred (see §4.1). They are thus different spatially from random eddies that form spontaneously and detach in fully turbulent flows [51].

#### 3.2.1. Flow with particles (two dimensions)

In order to investigate these eddies further we now introduce particle tracers, as proxies for crystals, into the flow simulations. A maximum of 10^4^ particles were used in the simulations. Particles can be introduced arbitrarily into the model at any position where they then track the mean velocity of the flow for the duration of the simulation. By assigning given particles a mass density (units of kg m^−3^), we can equate them to crystals that comprise the Basement Sill (table 1). In this way, the model is ‘tuned’ to the observed mineralogy of the intrusion through the known densities of the major mineral phases plagioclase and orthopyroxene.

#### 3.2.2. Single density particles

**Figure 9.**
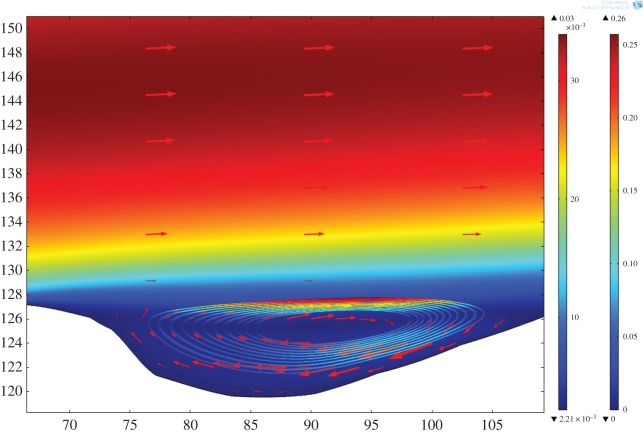
Eddy structure formed at the lower margin of the Sill. Streamlines define the particle orbital trajectory (density 2200 kg m^−3^); arrows show fluid motion. Magma viscosity 10 Pa s, density 2500 kg m^−3^.

Figure 9 shows what happens when particles of the same density (2200 kg m^−3^), but lighter than the suspending medium, are added to the flow in the vicinity of an undulation. Even this most simple case is instructive. Their distribution in the flow is not random. Instead the particle motion follows that of the streamlines in the eddy where collectively they trace out an orbital motion. The particles are trapped in the flow at a point corresponding to the eddy. The flow velocity in the eddy is considerably slower than the main flow above (arrows are proportional to the magnitude of the flow velocity). However, a new level of detail emerges within the eddy in the form of an orbital velocity gradient with speeds fastest at the top of the eddy and slowest on either side.

While not entirely unexpected given eddies are the result of boundary friction so that the upper eddy surface layer is coupled more closely to the faster-moving flow above, by extrapolation crystals with similar densities trapped in orbits inside a magma eddy will speed up and slow down as they circulate. Such non-uniform orbital velocities are unexpected. While beyond the scope of this analysis, there will doubtless be chemical implications arising from the mechanical effects of trapped crystal populations orbiting within the same intrusion but isolated from the main magma body.

#### 3.2.3. Multiple particles of varying density

**Figure 10.**
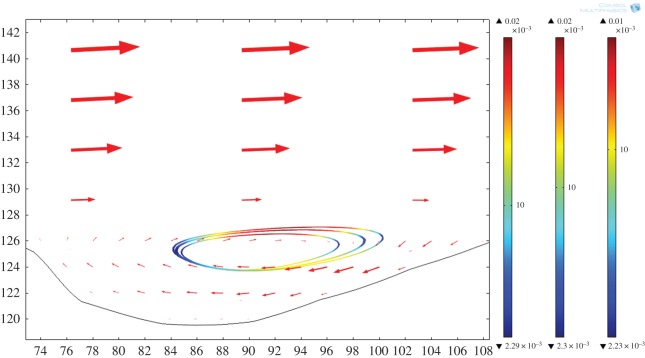
Multiple orbits for three different particle densities that correlate with the major mineral phases comprising the Basement Sill Lower Zone. Inner, 2000 kg m^−3^ (reference); mid, 2500 kg m^−3^ (plagioclase); outer, 3000 kg m^−3^ (orthopyroxene). Colour bars show relative rotational velocity of particles and magma. Magma viscosity, 10 Pa s, particle size 10 mm. Arrows show surface fluid velocity field.

Where particles of more than one density are considered, a segregation effect is observed in the particle orbits. This is shown in figure 10 where three populations of particles with densities ranging from 2000 to 3000 kg m^−3^ are analysed simultaneously. Again for simplicity melt viscosity (10 Pa s) and density are kept constant in the model to allow comparison with the single particle density simulation. Several interesting points of detail emerge. One is that we observe a segregation effect in particle orbits that is position-dependent. The orbits develop an asymmetry upstream of the flow with the highest density particles (3000 kg m^−3^) on the outside of the eddy and the least dense on the inside. It appears that circulation has separated particles out according to density, which follow rotational orbits that in two dimensions do not cross. Furthermore, each particle is orbiting at a different relative velocity. For the Basement Sill mineralogy, this implies that an initial mixture of Opx and plagioclase would segregate such that the denser Opx migrates to the outer regions of the eddy while less dense plagioclase is confined mostly to inner orbits, so long as circulation is maintained. The same effect is seen for particles with densities up to 5000 kg m^−3^. Finally, it appears from visual inspection that the asymmetry in segregation effect, with orbits stretched out in the downstream direction, is controlled by the slope of the Sill–country rock contact.

#### 3.2.4. Eddy formation and magma viscosity

**Figure 11.**
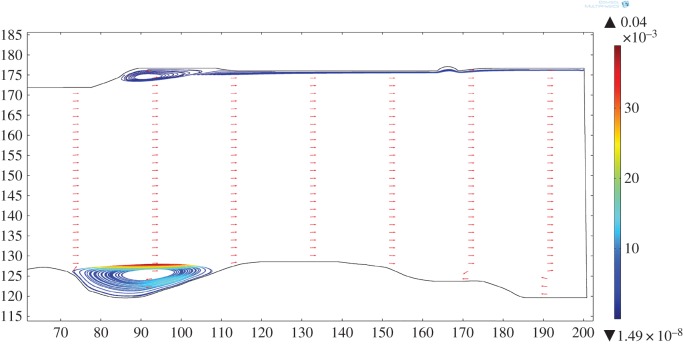
Extended view of model showing eddies at top and bottom of the intrusion. Single particle, density 2500 kg m^−3^ (plagioclase) magma viscosity 10 Pa s. Note some particles escaping from upper eddy back into the flow. Arrows show fluid velocity field. Reynolds number = 5800.

**Figure 12.**
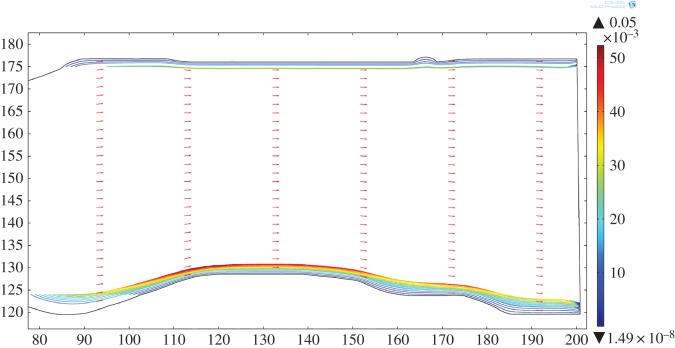
Simulation geometry as in figure 10 but with magma viscosity 10^3^ Pa s. Streamlines show particle trajectories. No eddy formation. Reynolds number ∼ 0.60.

In addition to overall flow velocities for the Basement Sill magma, the model allows us to examine the effect of increasing magma viscosity on eddy formation itself. Figure 11 shows eddy formation with particle orbits at two positions (base and roof) of the Sill for magma with a viscosity of 10 Pa s. At higher magma viscosities (more than 10^2^ Pa s), the eddy structures no longer appear in the simulations. Figure 12 shows the case for a magma viscosity of 10^3^ Pa s corresponding to the geometry in question. Again the particle streamlines follow a linear path with no closed orbits observed at any boundary undulation, implying a dampening effect of increasing magma viscosity on eddy formation. But the picture may be more complicated. Intuitively, there should be an effect because of the inverse relationship between viscosity and flow velocity and link to the Reynolds number as an overall indicator of non-laminar flow [51]. This idea is developed further in §4.1.

#### 3.2.5. Flow with particles (three dimensions)

**Figure 13.**
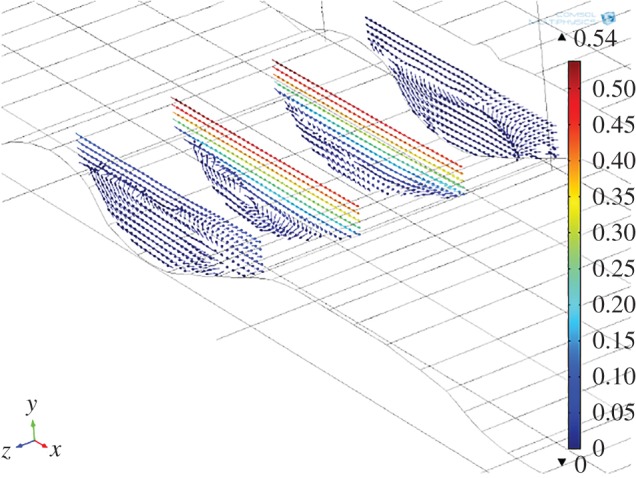
Three-dimensional simulation of eddy flow (without particles). Slices = volume velocity field (m s^−1^). Note velocity gradient in eddy rotation across the flow. Magma viscosity = 1 Pa s.

**Figure 14.**
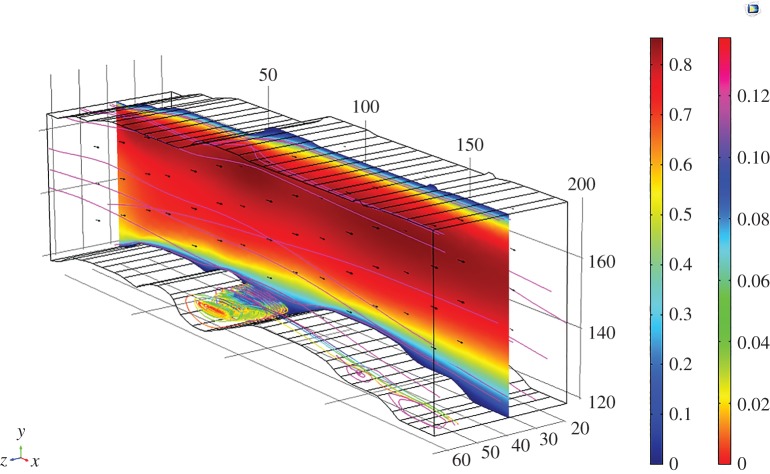
Larger-scale simulation of Sill for an arbitrary thickness (*z*) of 60 m and single density particles of 3000 kg m^−3^. Surface (m s^−1^) velocity magnitude (inner scale bar), particle velocity streamlines (outer scale bar). Note the roll-like geometry of particle orbits in three dimensions. Magma viscosity = 1 Pa s.

**Figure 15.**
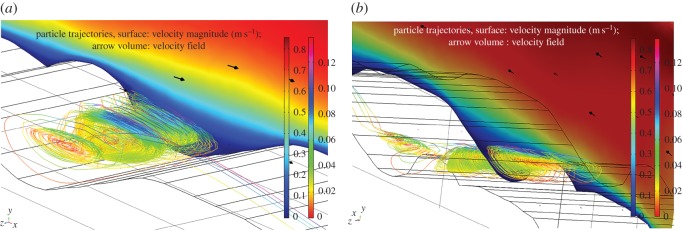
Detail of three-dimensional particle orbits in eddy (cf. figure 14). Single particle density 3000 kg m^−3^ (Opx), size 10 mm, magma density 2500 kg m^−3^, viscosity 1 Pa s. (*a*) Roll-geometry is clearly defined by the particle streamlines which show cross-stream variation in rotational velocity. Note some particles ejected back into the main flow. (*b*) Same eddy viewed from a different perspective shows how the particle orbital geometry extends across the Sill floor normal to the main flow field (shown as a two-dimensional slice). The model eddy structures correspond to a field scale of 1–10 metres.

The effect of geometry on magmas of the Basement Sill, in particular, its irregular contact with country rock in controlling local crystal segregation in low viscosity eddies is now apparent. The final part of our analysis has been to extend the model into the third dimension. This is speculative given there is no information about the dimension of the intrusion normal to the plane of intrusion (*z*) in the field at this location. In order to gain an impression of the flow dynamics in the vicinity of a magma eddy, we have extended the model arbitrarily to an upper width of *z* = 60 m. In reality, it is likely to be much wider. Figure 13 shows individual two-dimensional slices thorough the three-dimensional volume velocity field in the region of an undulation. The swirling nature of the fluid rotation is apparent, as is a cross-stream variation in rotation velocity. Figure 14 shows the effect of adding particles (crystals) to the simulation. The image comprises a vertical slice showing the magma velocity field along strike of the sill over a distance of approximately 300 m. Also shown is the three-dimensional form of particle streamlines caught in the eddy of figure 13. Eddies forming along the floor (and roof) of the intrusion have a three-dimensional geometry that is roll-like, extending at right angles into the flow. This is seen clearly in figure 15*a,b*.

While this example in relation to the Basement Sill is contrived due to the artificial nature of the imposed width in *z*, the internal complexity of eddies in three-dimensions poses interesting questions about how crystals might mix and interact during flow of this kind. For example, there is significant initial isotopic heterogeneity in the Basement Sill indicating crystals have grown in an open system (variable ^87^Sr/^86^Sr) and mechanically transported and juxtaposed [53]. It is thus possible that mixing of crystal populations trapped in slowly rotating, low-melt-viscosity eddies may help contribute to local chemical and isotopic diversity in some magmas [54].

## 4. Discussion

Our aim so far has been to show how image-based numerical modelling has the potential to augment field and experimental observations to better understand the intrusion and emplacement process. Because the input data cover a range of length scales, from grain size and density to the actual shape and dimensions of the Sill itself, a single suite of models can range freely from a micro (eddies and layering) to macro analysis of magma flow. In what follows we speculate on the wider geological implications of the flow model and the emplacement of the Ferrar magmas more generally.

### 4.1. Eddies and Reynolds numbers

So far we have drawn on a benchmark example of low viscosity magma (1 Pa s) as this captures eddy formation well given the model parameters (see also figure 11 for the 10 Pa s case). But is this value, lower than the calculated initial melt viscosity (table 1), overestimating the eddy effect in the model? Calculated Reynolds numbers (Re) are instructive here. For a melt viscosity of 33 Pa s, Re = *uL*/*ν*, where the kinematic viscosity *ν* = *η*/*ρ*, *L* is the intrusion width (thickness) in two dimensions and *u* the magma velocity (table 1). Taking the maximum model width of the Sill (80 m), Re = 1055, a value transitioning towards instability where the critical Reynolds number is approximately 2000 above which inertial forces become dominant [51]. Using the maximum observed Sill thickness *L* = around 300 m [27] gives Re = around 4000. Thus, for a melt viscosity of 33 Pa s we can expect instability to develop at undulose contacts between the Sill and country rock where the intrusion thickness exceeds approximately 200 m.

**Figure 16.**
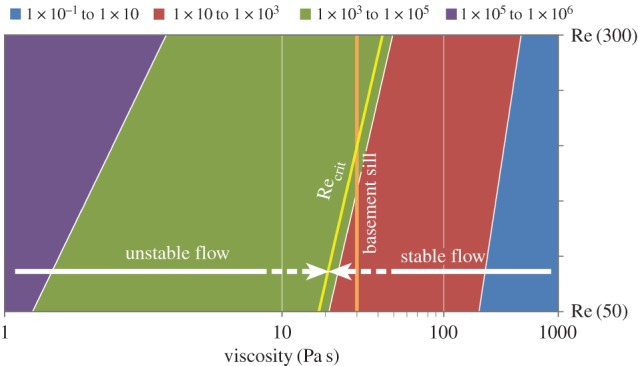
Domain map for modelled Basement Sill magmas (1 < *η* < 10^4^ Pa s) as a function of along-strike variation in Sill within the Wright Valley region (50–300 m, [27]). Reynolds numbers (10^−1^ < Re < 10^6^) scale with intrusion thickness and magma viscosity. Vertical line shows calculated melt viscosity of the Basement Sill for reference. Flow instability (static eddy formation) is predicted at uneven contacts between Sill and country rock where Re_crit_ > around 2000 (this model and [51]). For a melt viscosity of approximately 30 Pa s (Basement Sill) this corresponds to a Sill thickness of approximately 200 m.

Figure 16 shows the relationship between magma viscosity and Reynolds number for two end-member length scales of 50 and 300 m that define the variation in along-strike width of the Basement Sill in the Bull Pass region. With reference to figure 11 (§3.2.4), for a melt viscosity of 10 Pa and width of 80 m, Reynolds = 5800, increasing to 2 × 10^4^ for *L* = 300 m. However, for the higher viscosity case (10^3^ Pa), Re = 2.2 at maximum *L* = 300 m meaning viscous forces dominate. The zone defining the onset of flow instability appears to lie between magma viscosities of 10 and approximately 50 Pa s where, depending upon the intrusion characteristic length scale, eddy formation can in theory occur.

### 4.2. Eddies: thermal constraints

An important element missing from this analysis is temperature. Thus, the mechanical particle orbits described here differ from thermally induced convection effects in suspensions heated from above or below [55–58]. As eddies are a class of forced convection, then their cooling will not be exclusively by conduction. However, as an illustration, a characteristic (conductive) cooling time can be obtained from *t* = *l*^2^/*κ*, where *l* is the layer thickness and *κ* the thermal diffusivity (approx. 1 × 10^−6^ m s^−2^ in silicate melts [41]). Taking an eddy thickness of 10 m based on undulation wavelengths in the Basement Sill gives *t* = around 13 years. This is a short time corresponding to approximately 3% of the overall cooling time of the Sill of approximately 500 years [29] once motion has ceased. The complication of course is that so long as the flow is moving faster than heat is diffusing and magma supply is continuous, feedback between the thermal boundary layer close to the intrusion contact maintains an equilibrium allowing flow to proceed unhindered [41]. Indeed, as pointed out by Hamilton [27], the sheer scale and extent of the Antarctic sills, combined with their near uniform thickness, is evidence itself of magma capable of travelling a significant distance without cooling.

In summary, and by extrapolation, eddies in mafic flows with favourable Reynolds numbers, will be localized features limited in life by the destructive effect of increasing viscosity. Given the constraints and uncertainties involved in the model, predictions on their formation must be treated with caution. Nonetheless, their theoretical presence early in the life of low viscosity (less than or equal to 50 Pa s) melts may have potential to affect local composition through a style of orbital segregation not previously described.

### 4.3. Layering in the lower-zone websterite

**Figure 17.**
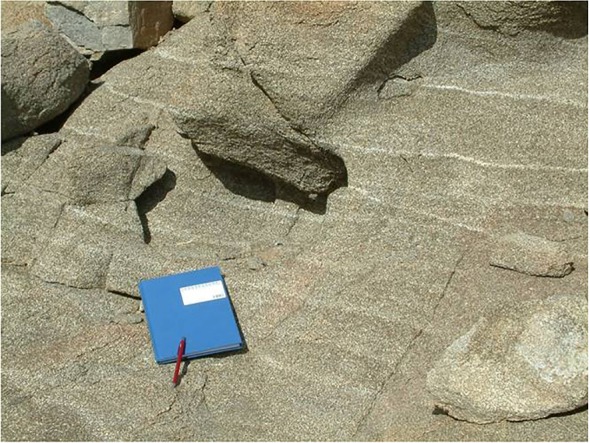
Small scale, rhythmic layering in the LZ websterite comprising thin bands of plagioclase dispersed within a predominantly Opx matrix. Model-derived shear strain rates for congested magma where crystal loads exceed approximately 30% (figure 8 and table 1) are comparable with experimental evidence for shear-thinning in congested magmas [33,35]. The implication is that the feldspar-rich layers formed by dilation in the magma slurry during emplacement [29,59].

The model also allows us to say something about the formation of internal structures separate to eddy development but a nonetheless persistent feature of the intrusion. Igneous layering in the Basement Sill [31], in particular, the origin of feldspathic bands in the LZ websterite has been investigated recently from the perspective of a magma intruding slurry [18,29,32,59]. The idea put forward is that feldspathic schlieren found in coarser grained regions of the Sill (figure 17) reflect segregation zones formed during granular flow of the congested magma (see also [12]). These planar structures differ from other types of segregation including vertical pipes and channels that formed during the later stages of Sill emplacement [29].

While the numerical model cannot predict the occurrence of crystal distributions comprising the layers, it does calculate the shear rate $\dot{\gamma }$. This important variable links magma viscosity to the shear stress. Results for magma viscosities relevant to slurries (0.3 < *ϕ* < 0.7) predict shear rates of approximately 10^−3^–10^−5^ s^−1^ (figure 8 and table 1) that overlap with experimental results on non-Newtonian (shear-thinning) magmas with solid fractions between 0.5 and 0.7 [33–36]. A deep characteristic of shear-thinning slurries is that they develop internal layering [52]. The order-of-magnitude melt flow velocity (*v*) in a mush undergoing shear can be found from $v=\dot{\gamma }hG\text{/}\theta ,$ where $\dot{\gamma }$ is the shear strain rate (obtained from the model), *h* is the layer height, *G* is the shear modulus of the major solid phase (Opx) and *θ* the mush strength [60]. Assuming values of *G* = 10^9^ Pa, *θ* = 10^8^ Pa [52] and a strain rate of 10^−4^ s^−1^, corresponding to a congested magma 56% by solids (table 1), melt segregation velocities are of the order of 0.01 cm s^−1^ over a vertical distance of 10 cm, a rough estimate of spacing between feldspathic layers (figure 17). Thus, dilatant pore fluid flow occurs on a timescale far in excess of the conductive cooling time, meaning the preservation potential of layers that are mechanical in origin should be high [29]. Indeed, it could be argued layering in congested magmatic slurries is an internal constraint, meaning its formation is an inevitable consequence of the intrusion process itself.

### 4.4. Long-range lateral flow

The final motivation of the numerical simulations was to obtain estimates of magma emplacement times. Assuming a well-insulated magma where the supply of melt is constant, recovered flow velocities can be used to help constrain models of lateral magma transport in the continental crust [61]. Geologists in Scott's 1901–1904 Discovery expedition and Shackleton's Nimrod expedition (1907–1909) noted an abundance of dolerite sills between Victoria Land and the Beardmore Glacier, a distance of some 800 km. Dolerite sills in East Antarctica crop out over an area of at least 5000 km^2^ and maintain a relatively consistent thickness, reflecting their high fluidity. As speculated by Hamilton [27], during intrusion on this scale, thousands of square kilometres of roof rock ‘floated’ above the sill complexes during emplacement, which link at scale to the surface Kirkpatrick flood basalts (figure 4).

The intrusive episode is likely to have lasted less than 1 Myr [28]. The Basement Sill itself extends at least 100 km and possibly much further [25]. Previous geochemical work on the Ferrar dolerites [62] in the Transarctic Mountains has made a strong case for significant lateral transport of magma extending more than 3000 km. Moreover, chemical data suggesting these magmas originated from a single source appears commonplace in the overall context of the Ferrar Large Igneous Province [63].

Assuming again a magma supply to support continuous flow, then from the modelling outcomes it is possible to place upper limits on lateral transport times for the Basement Sill magmas. So, at a constant viscosity of 33 Pa s, 3000 km could, in principle, be traversed in approximately 1 year. At higher magma viscosity (10^4^ Pa s), the same distance could be covered in less than 2 × 10^5^ years as chemically coherent magmas in sills [62,63], provided flow is continuous and uniform.

We end on what may seem an outlandish claim—that magma emplacement could be influenced by the rotation of the Earth. Magma is of course far more viscous than air or water meaning that compared to the atmosphere or oceans, any non-inertial effect is negligible on a local scale. Except in this case, magmatism is occurring on a planetary scale. To test this idea, we first estimate the Coriolis force ( *f*_c_ = 2*ω*sin(*φ*)), where 2*ω* is the Coriolis parameter (table 1), *φ* is latitude and *u* is the fluid velocity [37]. The Ferrar dolerites were emplaced 180 Ma when from palaeomagnetic evidence Antarctica was part of a landmass at approximately 58° S [27]. So, taking the calculated flow velocity for a 33 Pa s magma returns a small but finite result: *f*_c_ ∼ 1.42 × 10^−5^ m s^2^. Is this meaningful in the context of magma emplacement in a rotational frame of reference? Possibly, given the scale of LIPs in general (greater than 1 × 10^5^ km^2^) and their geologically short emplacement times [38]. By analogy, considerations into the effects of the Earth's rotation on the deflection of rivers and streams (Baer's Law) in relation to the Coriolis force [64] suggest there may too be a small effect. The non-dimensional Rossby Number, Ro = *u*/*f*_c_*l*, where *l* is the length of the flow, taken here as 3000 km, provides a cross check. A small number (≪1) for Ro identifies rotation as an important factor in the fluid system. For the Basement Sill, Ro = 7 × 10^−3^, implying a potential deflection effect over long distances, should the magma viscosity remain low. Whether this result could be detected geologically in the field is unlikely and anyway has probably never been looked for. However, we should not be surprised that continental-scale magmatic events are coupled in some small way to planetary rotation.

## 5. Summary

The Basement Sill complex, Antarctica, provides a world-class example of pervasive lateral flow of magma on a continental scale. In detail it offers an unprecedented two- and three-dimensional section through the frozen remnants of a congested magma slurry. Using a novel image-based numerical model where the intrusion geometry itself defines a bespoke finite-element mesh, we have simulated the dynamical (non-thermal) aspects of the flow regime over four orders of magnitude in viscosity. The model predicts the formation of magma eddies along the undulating base and roof of the intrusion. Eddies are a transient feature confined to low viscosity (less than or equal to 50 Pa s) melts, where inertial forces dominate. Numerical particle tracing, where particles are assigned density values corresponding to the Sill mineralogy, shows eddies can either trap particles (i.e. crystals) in orbit or eject them back into the flow at a later time according to their mass density. The isolation of crystals in this way may represent a hitherto unrecognized style of magmatic fractionation. We review the conditions necessary for shear-thinning in congested magma and argue that some of the magmatic layering in the LZ websterites comprising the Opx tongue is due to particle-melt segregation in the sheared mush during emplacement. Finally, the model is used to place lower bounds on lateral transport times for the Sill and by extension the Ferrar magmas. Under (ideal) conditions including uninterrupted magma supply and constant viscosity, it is feasible to fill the Basement Sill on a maximum timescale of approximately 10^5^ years. In theory, magmas emplaced rapidly during LIP formation may be in small part affected by the rotation of the Earth.

## Data accessibility

Image files and other data are deposited at Dryad (http://dx.doi.org/10.5061/dryad.sn69s) [65]. In addition, the model code and solution files are available at COMSOL Access Model Exchange https://www.comsol.com/community/exchange/491/.

## References

[1] Shaw HR 1965 Comments on viscosity, crystal settling and convection in granitic magmas. Am. J. Sci. 263, 120–152. (doi:10.2475/ajs.263.2.120)

[2] Shaw HR 1972 Viscosities of magmatic silicate liquids: an empirical method of prediction. Am. J. Sci. 272, 870–893. (doi:10.2475/ajs.272.9.870)

[3] Bottinga Y, Weill DF 1972 The viscosity of magmatic liquids: a model for calculations. Am. J. Sci. 272, 438–475. (doi:10.2475/ajs.272.5.438)

[4] McBirney AR, Murase T 1984 Rheological properties of magmas. Annu. Rev. Earth Planet Sci. 12, 337–357. (doi:10.1146/annurev.ea.12.050184.002005)

[5] Giordano D, Russell KJ, Dingwell D 2008 Viscosity of magmatic liquids: a model. Earth Planet. Sci. Lett. 271, 123–134. (doi:10.1016/j.epsl.2008.03.038)

[6] Marsh DB 1996 Solidification fronts and magmatic evolution. Min. Mag., 60, 5–40. (doi:10.1180/minmag.1996.060.398.03)

[7] Petford N 2003 Rheology of granitic magmas during ascent and emplacement. Annu. Rev. Earth Planet. Sci. 31, 339–427. (doi:10.1146/annurev.earth.31.100901.141352)

[8] Sumita I, Manga M 2005 Rheology of suspensions and the emplacement of tongues of crystal-rick magma within sills In *American Geophysical Union Meeting*, San Francisco. abstract V23A-0678.

[9] Cordonnier B, Hess K-U, Lavallee Y, Dingwell DB 2008 Rheological properties of dome lavas: case study of Unzen volcano. Earth Planet. Sci. Lett. 279, 263–272. (doi:10.1016/j.epsl.2009.01.014)

[10] Cimarelli CA, Costa A, Mueller S, Mader HM 2011 Rheology of magmas with bimodal crystal size and shape distributions: insights from analog experiments. Geochem. Geophys. Geosyst. 12, Q07024 (doi:10.1029/2011GC003606)

[11] Chevrel MA, Platz T, Hauber E, Baratoux D, Lavallée Y, Dingwell DB 2013 Lava flow rheology: a comparison of morphological and petrological methods. Earth Planet. Sci. Lett. 384, 109–120. (doi:10.1016/j.epsl.2013.09.022)

[12] Petford N 2009 Which effective viscosity? Min. Mag. 73, 167–191. (doi:10.1180/minmag.2009.073.2.167)

[13] Mader HM, Llewellin EW, Mueller SP 2013 The rheology of two-phase magmas: a review and analysis. J. Volc. Geotherm. Res. 257, 135–158. (doi:10.1016/j.jvolgeores.2013.02.014)

[14] Bergantz GW, Schleicher JM, Burgisser A 2015 Open-system dynamics and mixing in magma mushes. Nat. Geosci. 8, 793–796. (doi:10.10-38/NGEO2534)

[15] Wiebe RA, Collins WJ 1998 Depositional features and stratigraphic sections in granitic plutons: implications for the emplacement and crystallization of granitic magma. J. Struct. Geol. 20, 1273–1289. (doi:10.1016/S0191-8141(98)00059-5)

[16] Weinberg RF, Sial AN, Pessoa RR 2001 Magma flow within the Tavares pluton, northeastern Brazil: compositional and thermal convection. Geol. Soc. Am. Bull. 113, 508–520. (doi:10.1130/0016-7606(2001)113<0508:MFWTTP>2.0.CO;2)

[17] Smith TO, Ashwal LD 2015 Evidence for multiple pulses of crystal-bearing magma during emplacement of the Doros layered intrusion, Namibia. Lithos 238, 120–139. (doi:10.1016/j.lithos.2015.08.019)

[18] Marsh DB 2004 A magmatic mush column Rosetta stone: the McMurdo Dry Valleys of Antarctica. EOS 85, 497–502. (doi:10.1029/2004EO470001)

[19] Taylor CA, Steinman DA 2010 Image-based modeling of blood flow and vessel wall dynamics: applications, methods and future directions. Ann. Biomed. Eng.. 38, 1188–1203. (doi:10.1007/s10439-010-9901-0) 20087775 10.1007/s10439-010-9901-0

[20] Anderson MP, Woessner WW, Hunt J 2015 Applied groundwater modelling: simulation of flow and advective transport, 2nd edn, 564 p Amsterdam, The Netherlands: Elsevier.

[21] Ferrar HT 1907 Report on the field geology of the region explored during the ‘Discovery’ Antarctic Expedition, 1901–04. Nat, Hist. 1, 1–100.

[22] Taylor TG 1922 The physiography of the Mcmurdo Sound and Granite Harbour Region, British Antarctic (‘Terra Nova’) Expedition, 1910–1913, 246 pp London, UK: Harrisons & Sons Ltd.

[23] Webb PN, McKelvey BC 1959 Geological investigations in South Victoria Land, Antarctica Part 1. Geology of Victoria Dry Valley. NZ J. Geol. Geophys. 2, 120–136. (doi:10.1080/00288306.1959.10431317)

[24] Denton GH, Sugden DE, Marchant DR, Hall BL, Wilch TI 1993 East Antarctic ice sheet sensitivity to Pliocene climatic change from a Dry Valleys perspective. Geografiska Annaler 75A, 155–204. (doi:10.2307/521200)

[25] Elliot DH, Fleming TH 2004 Occurrence and dispersal of magmas in the Jurassic Ferrar Large Igneous Province, Antarctica. Gondwana Res. 7, 223–237. (doi:10.1016/S1342-937X(05)70322-1)

[26] Gunn BM 1962 Differentiation in Ferrar dolerites, Antarctica. NZ J. Geol. Geophys. 5, 820–863. (doi:10.1080/00288306.1962.10417641)

[27] Hamilton W 1965 Diabase sheets of the Taylor Glacier region Victoria Land, Antarctica. Geol. Survey Prof. Paper 456-B.

[28] Heimann Al, Flemming THG, Elliot DH, Foland KA 1994 A short interval of Jurassic continental flood basalt volcanism in Antarctica as demonstrated by ^40^Ar/^39^Ar geochronology. Earth Planet Sci. Lett. 212, 19–41. (doi:10.1016/0012-821X(94)90029-9)

[29] Bedard J, Marsh BD, Hershum TG, Naslund HR, Musaka SB 2007 large scale mechanical redistribution of orthopyroxene and plagioclase in the Basement Sill, Ferrar Dolerites, Antarctica. J. Petrol. 48, 2289–2326. (doi:10.1093/petrology/egm060)

[30] Marsh BD 1981 On the crystallinity, probability of occurrence, and rheology of lava and magma. Geol. Soc. Am. Bull. 106, 1720–1737. (doi:10.1007/bf00371146)

[31] Gunn BM 1963 Layered intrusions in the Ferrar dolerites, Antarctica. Mineralogical Soc. America Spec. Paper 1, 124–133.

[32] Charrier AD, Marsh BD 2005 Sill emplacement dynamics: experimental textural modelling of a pulsing, cooling, particle-laden magma as applied to the Basement Sill McMurdo Dry Valleys, Antarctica. AGU Abs V23-A0686.

[33] Caricchi L, Burlini LK, Ulmer P, Gerya T, Vassalli M, Papale P 2007 Non-Newtonian rheology of crystal-bearing magmas and implications for magma ascent dynamics. Earth Planet. Sci. Lett. 264, 402–419. (doi:10.1016/j.epsl.2007.09.032)

[34] Deubelbeiss Y, Kaus BJP, Connolly JAD, Caricchi L 2011 Potential causes for the non-Newtonian rheology of crystal-bearing magmas. Geochem. Geophys. Geosyst. 12, Q05007 (doi:10.1029/2010GC003485)

[35] Pistone ML, Caricchi P, Ulmer L, Burlini P, Ardia E, Reusser F, Marone, Arbaret L 2012 Deformation experiments of bubble- and crystal-bearing magmas: rheological and microstructural analysis. J. Geophys. Res. 117, B05208 (doi:10.1029/2011JB008986)

[36] Moitra P, Gonnermann HM 2015 Effects of crystal shape- and size-modality on magma rheology. Geochem. Geophys. Geosyst. 16, 1–26. (doi:10.1002/2014GC005554)

[37] Stull RB 2000 Meteorology for scientists and engineers, 502 p USA: Brooks/Cole.

[38] Coffin MF, Eldholm O 1994 Large igneous provinces: crustal structure, dimensions, and external consequences. Rev. Geophys. 32, 1–36. (doi:10.1029/93RG02508)

[39] Cashman KV, Soule SA, Mackey BH, Deligne NI, Deardorff ND, Dietterich HR 2013 How lava flows: new insights from applications of lidar technologies to lava flow. Geosphere 9, 1664–1680. (doi:10.1130/GES00706)

[40] Delaney PT, Pollard DD 1982 Solidification of basaltic magma during flow in a dike. Am. J. Sci. 282, 856–885. (doi:10.2475/ajs.282.6.856)

[41] Bruce PM, Huppert HE 1990 Solidification and melting along dykes by the laminar flow of Basaltic magma. In Magma transport and storage (ed. Ryan MP), pp. 87–101. New York, NY: John Wiley and Sons.

[42] Lister JR, Kerr RC 1990 Fluid-mechanical models of dyke propagation and magma-transport. In Mafic dykes and emplacement mechanisms (eds Parker AJ, Rickwood PC, Tucker DH), pp. 69–80. Rotterdam, The Netherlands: Balkema.

[43] Petford N, English R, Williams R, Rogers N 2012 High temperature steady shear and oscillatory rheometry of basaltic melt. Geophys. Res. Abstracts 14, EGU2012-3685-1.

[44] Lister J 1990 Buoyancy-driven fluid fracture; similarity solutions for the horizontal and vertical propagation of fluid-filled cracks. J. Fluid. Mech. 217, 213–239. (doi:10.1017/S0022112090000696)

[45] Lister JR, Kerr RC 1991 Fluid-mechanical models of crack propagation and their application to magma-transport in dykes. J. Geophys. Res. 96, 10 049–10 077. (doi:10.1029/91JB00600)

[46] Fialko YA, Rubin AM 1998 Thermodynamics of lateral dike propagation: implications for crustal accretion at slow spreading mid-ocean ridges. J. Geophys. Res. 103, 2501–2514. (doi:10.1029/97JB03105)

[47] Macdonald R, Wilson L, Thorpe RS, Martin A 1988 Emplacement of the Cleveland Dyke: evidence from geochemistry, mineralogy and physical modelling. J. Petrol. 29, 559–583. (doi:10.1093/petrology/29.3.559)

[48] Green RG, Greenfield T, White RS 2015 Triggered earthquakes suppressed by an evolving stress shadow from a propagating dyke. Nat. Geosci. 8, 629–632. (doi:10.1038/NGEO2491)

[49] Kerr RC, Lister JR 1995 The lateral intrusion of silicic magmas into unconsolidated sediments: the Tennant Creek porphyry revisited. Austral. J. Earth Sci. 42, 223–224. (doi:10.1080/08120099508728193)

[50] Holness MB, Humphreyes MCS 2003 The Traigh Bhan na Sgurra Sill, Isle of Mull: flow localisation in a major magma conduit. J. Petrology 44, 1961–1976. (doi:10.1093/petrology/egg066)

[51] Tritton JD 1988 Physical fluid dynamics, 544 p Oxford, UK: Clarendon Press.

[52] Stickel JJ, Powell RL 2005 Fluid mechanics and rheology of dense suspensions. Annu. Rev. Fluid Mech. 37, 129–149. (doi:10.1146/annurev.fluid.36.050802.122132)

[53] Davidson JP, Jerram D, Petford N 2005 Using micro-isotopic approaches to evaluate the origin and emplacement mechanism of the Basement Sill, Dry Valleys, Antarctica In *AGU* Abstract V23A-0679.

[54] Cashman K, Blundy J 2013 Petrological cannibalism: the chemical and textural consequences of incremental magma body growth. Contrib. Mineral. Petrol. 166, 703–729. (doi:10.1007/s00410-013-0895-0)

[55] Marsh DB, Maxey MR 1985 On the distribution and separation of crystals in convecting magma. J. Volc. Geothermal. Res. 24, 95–150. (doi:10.1016/0377-0273(85)90030-7)

[56] Huppert HE 1991 Buoyancy-driven motions in particle-laden fluids In *John Miles 70th Birthday Symposium*, vol. 141–160.

[57] Martin D, Nokes RI 1988 Crystal settling in a vigorously convecting magma chamber. Nature 332, 534–536. (doi:10.1038/332534a0)

[58] Koyaguchi T, Hallworth MA, Huppert HE 1993 An experimental study on the effects of phenocrysts on convecting magmas. J. Volc. Geotherm. Res. 55, 15–32. (doi:10.1016/0377-0273(93)90087-8)

[59] Petford N, Jerram D, Davidson JD 2005 Slurry flow and structures formation in a magma mush: the Basement Sill, McMurdo Dry Valleys, Antarctica In *American Geophysical Union Meeting*, San Francisco, abstract V13H-05.

[60] Petford N, Koenders MA 2003 Shear-induced pressure changes and seepage phenomena in a deforming porous layer – I. Geophys. Res. Int, 155, 857–869. (doi:10.1111/j.1365-246X.2003.02076)

[61] Lister JR 1995 Fluid-mechanical models of the interaction between solidification and flow in dykes. In Physics and chemistry of dykes (eds Baer G, Heimann A), pp. 115–124. Rotterdam, The Netherlands: Balkema.

[62] Elliot DH, Fleming TH, Kyle PR, Foland KA 1999 Long distance transport of magma in the Jurassic Ferrar Large Igneous Province, Antarctica. Earth Planet. Sci. Lett. 167, 89–104. (doi:10.1016/S0012-821X(99)00023-0)

[63] Leat PT 2008 On the long-distance transport of Ferrar Magmas. In Structure and emplacement of high-level magmatic systems, vol. 302 (eds Thomson K, Petford N), pp. 45–61. London, UK: Geological Society of London. Geol. Soc. Spec. Pub.

[64] Einstein A 1954 The causes of the formation of meanders in the courses of rivers and of the so-called Baer's Law. In Ideas and opinions, 377 p New York, NY: Bonanza Books.

[65] Petford N, Mirhadizadeh S 2017 Data from: Image-based modelling of lateral magma flow: the basement sill, Antarctica. Dryad Digital Repository. (http://dx.doi.org/10.5061/dryad.sn69s) 10.1098/rsos.161083 PMC5451803 28573002

